# Lateral Ventricular Neural Stem Cells Provide Negative Feedback to Circuit Activation Through GABAergic Signaling

**DOI:** 10.3390/cells14060426

**Published:** 2025-03-13

**Authors:** Moawiah M. Naffaa, Henry H. Yin

**Affiliations:** 1 Department of Psychology and Neuroscience, Duke University, Durham, NC 27708, USA; 2 Department of Cell Biology, Duke University School of Medicine, Durham, NC 27710, USA; 3 Department of Neurobiology, Duke University School of Medicine, Durham, NC 27710, USA

**Keywords:** lateral ventricular neural stem cells (LV NSCs), anterior cingulate cortex-subependymal-ChAT^+^ (ACC-subep-ChAT^+^) circuit, GABAergic signaling, monoamine oxidase B (MAOB), SLC6A1, GABA transporter-1 (GAT-1), LRRC8D, volume-regulated anion channels (VRACs)

## Abstract

Recent studies have demonstrated that circuit activation in vivo can regulate proliferation of lateral ventricular neural stem cells (LV NSCs), although the underlying molecular and cellular mechanisms are not yet fully understood. Here, we investigated the role of GABAergic signaling in the interaction between LV NSCs and the anterior cingulate cortex-subependymal-choline acetyltransferase^+^ (ChAT^+^) neuron (ACC-subep-ChAT^+^) circuit. We found that monoamine oxidase B (MAOB), a key enzyme involved in gamma-aminobutyric acid (GABA) synthesis, is expressed in LV NSCs, and that activation of the ACC-subep-ChAT^+^ circuit can modulate MAOB activity. Additionally, LV NSCs express LRRC8D, a core component of volume-regulated anion channels, and GABA transporter-1 (GAT-1, SLC6A1). We show evidence that, through GABA signaling, LRRC8D and GAT-1 can provide a negative feedback signal to ChAT^+^ neurons, a key component of the ACC-subep-ChAT^+^ circuit that regulate proliferation of LV NSCs. These findings suggest that MAOB-driven GABA synthesis, LRRC8D-regulated chloride and GABA transport, and GAT-1-facilitated GABA reuptake can regulate neural circuit activation and influence NSC proliferation dynamics in the LV.

## 1. Introduction

The subventricular zone (SVZ) is one of the two principal neurogenic niches in the adult brain [1,2]. In rodents, neurons generated in the SVZ migrate to the olfactory bulb, where they differentiate into granule neurons and periglomerular neurons [3,4,5]. Upon integration into the olfactory bulb’s local circuitry, these neurons play a key role in olfaction and related behaviors [6,7,8].

Neural stem cells (NSCs) can be quiescent or active, and these states are regulated by many factors including synaptic inputs from various brain regions [9,10]. For example, NSCs in the lateral ventricle (LV) are influenced by projections from the hypothalamus and cingulate cortex [11]. Previous work showed that the anterior cingulate cortex (ACC), an area in the frontal cortex, projects to subependymal-neurons expressing choline acetyltransferase (ChAT^+^) bordering the LV, and this ACC-subep-ChAT^+^ circuit modulates the activity of quiescent LV NSCs through the activation of muscarinic M3 receptors [12,13]. M3 activation leads to intracellular calcium release from the endoplasmic reticulum, which initiates a signaling cascade that ultimately promotes proliferation in LV NSCs [13]. This finding raises the question of whether and how LV NSC proliferation may influence the ACC-subep-ChAT^+^ circuit. In particular, can the NSCs produce a negative feedback signal that reduce circuit activation? One potential mechanism is gamma-aminobutyric acid (GABA)-mediated signaling, as subep-ChAT^+^ neurons also express GABA-A receptors [12]. We hypothesize that LV NSCs can synthesize GABA and/or uptake it from the extracellular space and release it into the extracellular environment and inhibit subep-ChAT^+^ neurons. This mechanism may serve as negative feedback that regulates ACC-subep-ChAT^+^ neuronal activity on LV NSCs and ultimately modulates LV NSC proliferation.

To test this hypothesis, we investigated the capacity of LV NSCs in the ventral SVZ to synthesize and regulate GABA transport. We examined whether LV NSCs express the necessary enzymes or channels for GABA synthesis, release, and reuptake, how ACC-subep-ChAT^+^ circuit activity influences GABA signaling in LV NSCs and its presence in the extracellular space, and whether pharmacological modulation of these transporters and receptors involved in GABA uptake or release affects circuit activity and the regulation of LV NSC proliferation.

We first analyzed published RNA sequencing (RNA-seq) data from SVZ LV NSC cultures, including both control and carbachol-treated samples [14]. The RNA-seq data identified high expression levels of monoamine oxidase B (MAOB) in LV NSCs. MAOB, an enzyme predominantly found in astrocytes [15], catalyzes the breakdown of putrescine to facilitate GABA synthesis [16,17,18,19]. Furthermore, there is significant expression of LRRC8D, the largest isoform in the LRRC8 protein family. LRRC8D functions as a subunit of the volume-regulated anion channels (VRACs) [13], which may play a role in transporting GABA [20,21]. Finally, we also found significant expressions of SLC6A1, the gene encoding the GABA transporter 1 (GAT-1), which contributes to the reuptake of extracellular GABA into neurons and glia [22,23].

**Figure 1:**
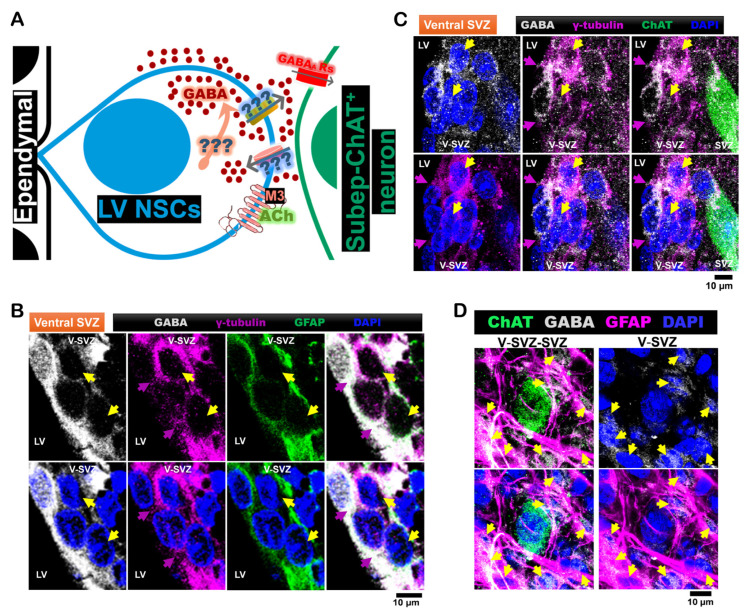
LV NSCs express GABA in the ventral subventricular zone, surrounding subep-ChAT⁺ neurons. (**A**). Schematic representation of the cellular composition and organization of the ventral LV-SVZ, highlighting ependymal cells, LV NSCs, transit-amplifying cells, and LV NSCs. (**B**). Immunofluorescence staining for GABA (gray), GFAP (green), and γ-tubulin (purple) in the ventral V-SVZ of P40 C57BL/6J mouse brain coronal sections. Yellow arrows indicate GABA⁺-GFAP⁺-γ-tubulin⁺ NSCs in the ventral V-SVZ, while purple arrows indicate apical γ-tubulin⁺ NSC endings in the ventral SVZ. Scale bar = 10 µm. (**C**). Immunofluorescence staining for GABA (gray), ChAT (green), and γ-tubulin (purple) in the ventral V-SVZ adjacent to subep-ChAT⁺ neurons of P40 C57BL/6J mouse brain coronal sections. Yellow arrows indicate GABA⁺-γ-tubulin⁺ NSCs in the ventral V-SVZ, while purple arrows indicate apical γ-tubulin⁺ NSC endings in the ventral SVZ. Scale bar = 10 µm. (**D**). Immunofluorescence staining for GABA (gray), ChAT (green), and GFAP (purple) in SVZ whole-mount preparations of C57BL/6J (P35) mice. Yellow arrows indicate GABA⁺-GFAP⁺ cells in the ventral V-SVZ surrounding subep-ChAT⁺ neurons. Scale bar = 10 µm. Images are representative of three mice.

We found that, in LV NSCs, MAOB expression is increased in response to ACC-subep-ChAT^+^ circuit activation. LRRC8D and SLC6A1 proteins are expressed in both quiescent and activated NSCs within the ventral SVZ. They appear to regulate the impact of ACC-subep-ChAT^+^ circuit stimulation on LV NSC proliferation. These findings suggest a potential role for GABA in providing negative feedback to the ACC-subep-ChAT⁺ circuit, which normally promotes the proliferation of LV NSCs (Figure 1A).

## 2. Materials and Methods

### 2.1. Chemicals

The compounds used in this study were as follows: BMP-4 Protein (Catalog #: 5020-BP, R&D Systems, Minneapolis, MN, USA), Carbachol (Catalog #: 212385-M, Millipore Sigma, Saint Louis, MO, USA), Compound 21 (DREADD Agonist 21 dihydrochloride, Catalog #: SML2392, Sigma-Aldrich. Saint Louis, MO, USA), DCPIB (Catalog #: 1540, Tocris, Bristol, UK), and CI966 (Catalog #: 1296, Tocris, Bristol, UK).

### 2.2. Mice

All experiments were conducted in compliance with an approved protocol from the Institutional Animal Care and Use Committee at Duke University, Durham, NC, USA. Mice were group-housed under a 12 h light/dark cycle (lights on at 7 a.m.), a controlled ambient temperature of 21 °C, and 45% humidity. The following mouse lines were obtained from The Jackson Laboratory (JAX, Bar Harbor, ME, USA): C57BL/6J (Stock #000664) and calretinin-Cre (Cr-Cre, Stock #010774). *CR-Cre* mice are used for in vivo chemogenetic experiments. ACC neurons that project to subep-ChAT^+^ neurons upregulate calretinin around age P28 [12]. Both male and female mice, aged between postnatal day 35 (P35) and postnatal day 65 (P65), were included in the study.

### 2.3. Viruses

pAAV-hSyn-DIO-hM3D(Gαq)-mCherry (Addgene #44361) was obtained from Addgene, Watertown, MA, USA. This vector enables selective neuronal activation via the hSyn promoter, ensuring neuron-specific expression, and a DIO system, which restricts transgene expression to CR-Cre-expressing neurons in the ACC, allowing precise circuit modulation. The hM3D(Gαq) DREADD receptor, a modified muscarinic receptor, is selectively activated by C21 (also known as cpd21), leading to neuronal depolarization and circuit activation.

### 2.4. Antibodies

Primary antibodies included Anti-Choline Acetyltransferase Antibody (Catalog #AB144P, Millipore Sigma, Saint Louis, MO, USA), Mash1 antibodies from ThermoFisher Scientific (Catalog #14-5794-82) and Abcam (Catalog #ab211327), and EGRF (Catalog #AF231, R&D Systems, Minneapolis, MN, USA). Additional primary antibodies used were GFAP Chicken Antibody (Catalog #GFAP, Aves Labs), γ-tubulin Monoclonal Antibody (4D11) (Catalog #MA1-850, ThermoFisher Scientific, Waltham, MA, USA), GABA Polyclonal Antibody (Catalog #PA5-32241, ThermoFisher Scientific, Waltham, MA, USA), Monoamine Oxidase B Polyclonal Antibody (Catalog #PA5-18442, ThermoFisher Scientific, Waltham, MA, USA), LRRC8D Polyclonal Antibody (Catalog #11537-1-AP, Proteintech), and SLC6A1 Polyclonal Antibody (Catalog #PA5-85766, ThermoFisher Scientific, Waltham, MA, USA). The antibodies (markers) used in this study to label quiescent and activated states of NSCs and subep-ChAT^+^ neurons are summarized in Table 1.

For secondary antibodies, we used Alexa Fluor^®^ 488 AffiniPure™ Donkey Anti-Rabbit IgG (H + L) (Catalog #711-545-152, Jackson ImmunoResearch, West Grove, PA, USA) and Alexa Fluor^®^ 594 AffiniPure™ Donkey Anti-Rabbit IgG (H + L) (Catalog #711-585-152, Jackson ImmunoResearch, West Grove, PA, USA). Additional reagents included Alexa Fluor^®^ 594 AffiniPure™ Donkey Anti-Goat IgG (H + L) (Catalog #705-585-003, Jackson ImmunoResearch, West Grove, PA, USA), Alexa Fluor^®^ 647 AffiniPure™ Donkey Anti-Goat IgG (H + L) (Catalog #705-605-147, Jackson ImmunoResearch, West Grove, PA, USA), and Alexa Fluor^®^ 488 AffiniPure™ Donkey Anti-Mouse IgG (H + L) (Catalog #715-545-150, Jackson ImmunoResearch, West Grove, PA, USA). Secondary antibodies targeting chicken IgY (IgG) included Alexa Fluor^®^ 488 AffiniPure™ Donkey Anti-Chicken IgY (H + L) (Catalog #703-545-155, Jackson ImmunoResearch, West Grove, PA, USA) and Alexa Fluor^®^ 647 AffiniPure™ Donkey Anti-Chicken IgY (H + L) (Catalog #703-605-155, Jackson ImmunoResearch, West Grove, PA, USA). Finally, Alexa Fluor^®^ 647 AffiniPure™ Donkey Anti-Mouse IgG (H + L) (Catalog #715-605-151, Jackson ImmunoResearch, West Grove, PA, USA) was also utilized.

### 2.5. Stereotaxic Injections

Stereotaxic injections were performed in mice secured in a stereotaxic frame (David Kopf Instruments) under isoflurane anesthesia. For in vivo chemogenetic activation of the ACC-subep-ChAT^+^ circuit, 300 nL of the viral vector (titer: 2.2 × 10^13^ GC/mL) pAAV-hSyn-DIO-hM3D(Gαq)-mCherry was injected into the ACC region of *Cr-Cre* (P30) mice. The injection was performed at the following coordinates relative to Bregma: anterior-posterior (AP) +0.8 mm, mediolateral (ML) ± 0.25 mm, dorsoventral (DV) −0.3 mm from the brain surface. Viral infusion was performed slowly for over 10 min using a Nanoject injector (Drummond Scientific, Broomall, PA, USA) connected to a glass pipette. To ensure optimal viral distribution, the pipette was left in place for an additional 10 min post-injection before being retracted.

Local inhibition of LRRC8D and SLC6A1 was achieved using DCPIB and CI966, respectively, using a micro-osmotic pump (Model 1003D, Alzet, Cupertino, CA, USA) for drug delivery. DCPIB (4-(2-butyl-6,7-dichloro-2-cyclopentyl-2,3-dihydro-1-oxo-1H-inden-5-yl)oxy]butanoic acid) is a potent and selective inhibitor of volume-regulated anion channels (VRACs). It effectively inhibits VRAC-mediated chloride ion (Cl^−^) currents at concentrations that do not significantly affect other chloride channels, such as the cystic fibrosis transmembrane conductance regulator (CFTR), ClC chloride channels, or calcium-activated chloride channels (CaCC) [24]. DCPIB is a potent inhibitor of VRAC-mediated conductance [25]. CI-966 (NNC-711; 1-(2-((Diphenylmethylene)amino)oxy)ethyl)-1,2,5,6-tetrahydro-3-pyridinecarboxylic acid) is a potent and selective inhibitor of γ-aminobutyric acid (GABA) uptake. It exerts its effects by targeting GABA transporter 1 (GAT-1) [26,27]. During the same surgical session, a cannula was implanted into the LV of Cr-Cre (P30) mice at the following coordinates: AP +0.8 mm, ML ± 0.65 mm, DV −2.1 mm from the brain surface. An amount of 300 nL of the pAAV-hSyn-DIO-hM3D(Gαq)-mCherry virus was injected into the ACC as previously described.

Cortical circuit activation was achieved via intraperitoneal administration of DREADD agonist C21 10 h after connecting the osmotic pump, which facilitated drug perfusion. The osmotic pump delivered the drugs at a flow rate of 1 µL per hour for a duration of 12 h, following the manufacturer’s instructions.

### 2.6. Immunofluorescence Staining and Imaging

Brain tissues were prepared for immunohistochemical (IHC) staining to investigate subep-ChAT^+^ neurons and cortical circuit modulations in chemogenetic in vivo experiments. At the conclusion of the experiments, mice were deeply anesthetized with isoflurane and transcardially perfused with phosphate-buffered saline (PBS), followed by 4% paraformaldehyde (PFA) in PBS. The brains were then carefully removed and post-fixed in 4% PFA at 4 °C overnight.

For SVZ whole-mount preparations, the dissected tissues were incubated in a blocking solution consisting of 5% donkey serum and TBST for 100 min at room temperature. Subsequently, they were incubated overnight at room temperature in PBS containing 1% donkey serum and specific primary antibodies validated either in our prior publication [12] or in vendor-provided references. Following primary antibody incubation, the tissues were washed in PBS and then treated with secondary antibodies, including Alexa-594 (1:1000), Alexa-488 (1:1000), or Alexa-647 (1:1000), for two hours at room temperature. Afterward, the tissues were washed in PBS and counterstained with 4′,6-diamidino-2-phenylindole (DAPI; Sigma-Aldrich, Saint Louis, MO, USA). Finally, the samples were washed four times with TBST and mounted on slides using Fluoromount (Sigma, Saint Louis, MO, USA), an aqueous mounting medium.

Images were acquired using a Leica SP8 upright confocal microscope equipped with 10×, 40×, and 63× objectives, controlled via LAS X Life Science Microscope software (LASX 3.10.0). To visualize the ventricular-SVZ (V-SVZ) region within the SVZ niche, tile-scan imaging was used, and confocal stacks of 1.5 to 3 μm optical sections were captured to illustrate the monolayer of cells just beneath the ependymal layer. The V-SVZ layer is labeled in the images presented in this manuscript. Additionally, wider-field images encompassing both the ventral SVZ and SVZ regions were acquired using confocal stacks ranging from 8 to 11 μm in depth. These images depict the spatial arrangement of subep-ChAT+ neurons within the SVZ and the layer of LV NSCs, including quiescent LV NSCs, located beneath the ependymal layer in the V-SVZ region.

### 2.7. In Vivo Stimulation of Chemogenetic Circuits and Local Protein Inhibition in the SVZ

**Figure 2:**
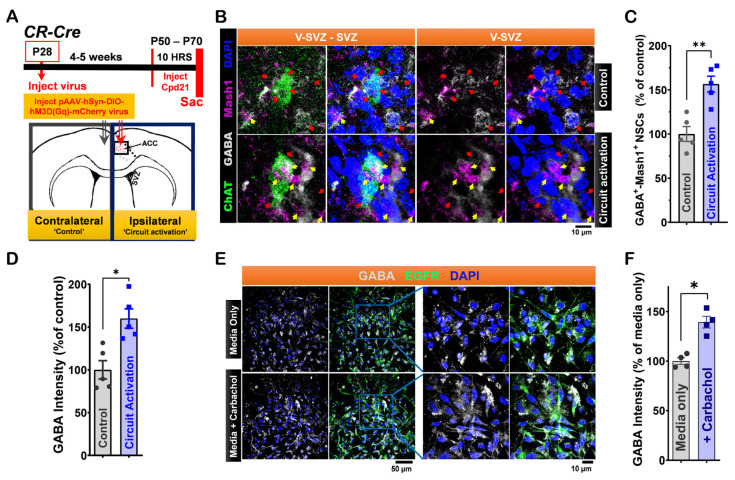
Modulation of LV NSCs via ACC-Subep-ChAT^+^ Circuit In Vivo and Carbachol Treatment In Vitro Alters GABA signaling. (**A**). Experimental design (upper) and schematic representation (lower) of in vivo chemogenetic activation for 10 h using the pAAV-hSyn-DIO-hM3D(Gαq)-mCherry virus injected into the ACC. (**B**). Representative immunofluorescence staining for GABA (gray), ChAT (green), and Mash1 (purple) in contralateral SVZ wholemounts (control, no chemogenetic activation of the ACC-subep-ChAT⁺ circuit) and ipsilateral SVZ wholemounts following chemogenetic circuit activation as described in (**A**). Yellow arrows indicate GABA⁺-Mash1⁺ NSCs, while red arrows indicate NSCs that are only GABA⁺. Scale bar = 10 μm. (**C**). Quantification of GABA⁺-Mash1⁺ NSCs relative to GABA⁺ NSCs per subep-ChAT⁺ neuron in contralateral and ipsilateral SVZ wholemounts from (**B**). ** *p* = 0.0020, t_4_ = 7.187, paired *t*-test. N = 5 CR-Cre mice. Each data point corresponds to the calculated mean percentage of GABA⁺-Mash1⁺ NSCs as a subset of the total GABA⁺ NSC population within a specified ROI. These ROIs were carefully delineated within the V-SVZ layer in proximity to subep-ChAT⁺ neurons (averaged from four subep-ChAT⁺ neurons per mouse). (**D**). Quantification of GABA intensity relative to GABA⁺ NSCs per subep-ChAT⁺ neuron in SVZ wholemounts from (**B**). * *p* = 0.0158, t_4_ = 4.023, paired *t*-test. N = 5 CR-Cre mice. Each data point in the analysis represents the mean percentage of GABA intensity measured specifically within GABA⁺ NSCs located in a defined ROI. ROIs were selected within the V-SVZ layer, in areas immediately surrounding subep-ChAT⁺ neurons (averaged from four subep-ChAT⁺ neurons per mouse). (**E**). Immunofluorescence staining for GABA (gray) and EGFR (green) in control or carbachol-treated (15 μM) SVZ NSC cultures collected after 24 h in proliferation media. Scale bar = 50 μm. (**F**). Quantification of GABA intensity relative to EGFR⁺ NSCs in control and carbachol-treated SVZ NSC cultures. * *p* = 0.0153, t₃ = 5.015, paired *t*-test. N = 4 independent SVZ NSC cultures per group. Each data point represents the average percentage of GABA intensity specifically within EGFR⁺ NSCs.

Following stereotaxic viral infusion as detailed in the “Stereotaxic Injections” section and the experimental design illustrated in Figure 2A, C21 (also known as DREADD Agonist 21 dihydrochloride or CPD21) was prepared by dissolving in 0.9% saline and stored at −20 °C until use. Intraperitoneal injections of C21 were administered at a dose of 1 mg/kg. Chemogenetic circuit stimulation in vivo was initiated by intraperitoneal C21 injection, delivered 6 h prior to the experiment’s conclusion [12,13].

Simultaneous assessments of in vivo chemogenetic circuit stimulation and localized inhibition of LRCC8D and SLC6A1 proteins in the LVs were conducted. In these experiments, the ventral SVZ was exposed to localized protein inhibition using DCPIB (5 µM) and CI966 (1.5 µM) via continuous infusion for 12 h. Circuit activation was initiated during the final 10 h.

SVZ wholemounts were dissected to examine the expressions of Mash1, GABA, MAOB, LRRC8D, and SLC6A1. Quantitative comparisons were made between the ipsilateral sides (receiving circuit activation or combined circuit activation and local protein inhibition) and the contralateral control sides. Specifically, the number of GABA^+^ Mash1^+^ NSCs/GABA^+^ NSCs, MAOB^+^ Mash1^+^ NSCs/MAOB^+^ NSCs, LRRC8D^+^ Mash1^+^ NSCs/LRRC8D^+^ NSCs, and SLC6A1^+^ Mash1^+^ NSCs/SLC6A1^+^ NSCs surrounding subep-ChAT^+^ neurons were analyzed. These comparisons provided insights into the spatial and functional effects of chemogenetic stimulation and the inhibition of LRRC8D and SLC6A proteins on neural stem cell populations.

### 2.8. SVZ NSC Culture

SVZ NSC cultures were derived from SVZ wholemounts obtained from postnatal (P12) C57BL/6J mice as described previously [13]. The dissected tissues were placed in DMEM/F-12 (DF) medium supplemented with 100 units/mL penicillin, 100 μg/mL streptomycin, and 250 ng/mL amphotericin B (abx). Following dissection, the pooled tissues were incubated in 0.005% trypsin at 37 °C (pH 7.3) for 15 min.

After enzymatic digestion, the tissues were transferred to uncoated T75 plastic tissue-culture dishes and incubated overnight in N5 medium. The N5 medium consisted of DF supplemented with N2 supplements, 35 μg/mL bovine pituitary extract, abx, 5% fetal calf serum (FCS, HyClone), and 40 ng/mL each of epidermal growth factor (EGF) and basic fibroblast growth factor (bFGF). Unattached cells were collected, replated on uncoated plastic dishes, and cultured in N5 medium until they reached 90–100% confluency. The medium was refreshed every other day for 3–4 days during proliferation.

Freshly cultured cells were used for all experiments. To induce quiescence in LV NSCs, cells were exposed to 10 ng/mL BMP4 for 24 h. Following quiescence induction, cells were treated under one of three conditions for an additional 24 h: media only (control), media supplemented with carbachol (15 µM), or media containing carbachol (15 µM) combined with a selective protein inhibitor (CI966). At the conclusion of the treatment period, experiments were terminated by fixing the cells in 4% PFA. Subsequent immunostaining was performed to analyze and quantify cellular responses.

### 2.9. Quantification and Statistical Analysis

Statistical analyses were performed using GraphPad Prism software (version 8). Paired *t*-tests were used for both in vivo chemogenetic stimulation studies and in vitro SVZ NSC culture modulation experiments.

To quantify the proportions of GABA⁺-Mash1⁺ NSCs/GABA⁺ NSCs, MAOB⁺-Mash1⁺ NSCs/MAOB⁺ NSCs, LRCC8D⁺-Mash1⁺ NSCs/Mash1⁺ NSCs, and SLC6A1⁺-Mash1⁺ NSCs/SLC6A1⁺ NSCs, as well as the average intensities of GABA per GABA⁺ NSC, MAOB per MAOB⁺ NSC, and SLC6A1 per SLC6A1⁺ NSC in the V-SVZ, 50 μm × 50 μm square images were captured. Each image of the V-SVZ layer (comprising consecutive two-dimensional confocal stacks covering the entire V-SVZ) was centered on a subep-ChAT⁺ neuron to facilitate the quantification of LV NSCs and the analysis of NSC proliferation activity surrounding these neurons.

Biological replicates are stated in the figure legends (e.g., N = 5 mice). Paired *t*-tests were performed to compare the ipsilateral and contralateral sides of the brain within the same animal. The mean number of stained NSCs was calculated from five subep-ChAT⁺ neurons on both the ipsilateral and contralateral sides for each mouse.

Additionally, the quantification of SLC6A1⁺-EGFR⁺ NSCs, as well as the average intensities of GABA per EGFR⁺ NSC and SLC6A1 per EGFR⁺ NSC, was done using data from four independent SVZ NSC culture experiments (N = 4). For each experiment, 2–3 wells were analyzed, and paired *t*-tests were used to assess statistical significance.

## 3. Results

### 3.1. Expression and Regulation of GABA Activity in LV NSCs by the ACC-Subep-ChAT⁺ Circuit Activation and Cholinergic Stimulation

Based on RNA-seq data, we hypothesized that LV NSCs possess molecular machinery for synthesizing GABA via MOAB and regulating its transport through LRRC8D and GAT-1. First, we measured GABA expression in brain tissues from P40 C57BL/6J mice. Coronal brain sections were immunostained for GABA, glial fibrillary acidic protein (GFAP), and γ-tubulin to investigate the ventral SVZ domain. GFAP was used as a marker for both quiescent and active LV NSCs, while γ-tubulin specifically identified quiescent NSCs. We found that GFAP⁺-γ-tubulin⁺ cells, corresponding to quiescent LV NSCs, were also positive for GABA (Figure 1B). We also confirmed that quiescent LV NSCs near subep-ChAT⁺ neurons in the ventral SVZ. were GABA⁺ and γ-tubulin⁺ (Figure 1C). In whole-mount preparations from P35 C57BL/6J mice, GFAP⁺ LV NSCs in the ventral SVZ domain were also GABA⁺, confirming GABA expression in LV NSCs that are close to subep-ChAT⁺ neurons (Figure 1D).

Prior to examining circuit modulation of GABA activity in LV NSCs in the V-SVZ, we investigated potential differences in GABA expression across developmental stages (P30 and P55), between hemispheres at P55, and between sham and control groups at P55. First, we analyzed GABA intensity within GABA⁺-GFAP⁺ NSCs in the V-SVZ surrounding subep-ChAT⁺ neurons in mice at P30 and P55. Our findings revealed no significant differences between these age groups (Figure S1). Additionally, we examined GABA intensity in GABA⁺ cells surrounding subep-ChAT⁺ neurons in the ventral SVZ of P55 mice, comparing the two hemispheres to assess potential interhemispheric variability. The results indicated no significant differences in GABA intensity between the two hemispheres (Figure S2A,B). Furthermore, we compared GABA intensity in GABA⁺ cells within the V-SVZ region surrounding subep-ChAT⁺ neurons in P55 *CR-Cre* mice between the ipsilateral side injected with the pAAV-hSyn-DIO-hM3D(Gαq)-mCherry virus at P28 and the uninjected contralateral ‘control’ side. The results demonstrated no significant differences in GABA activity between the sham and control sides (Figure S2C,D). Collectively, these findings indicate that GABA expression remains stable between P30 and P55, with no significant interhemispheric differences at P55.

To examine circuit modulation of GABA activity in LV NSCs, we used chemogenetics to activate the ACC-subep-ChAT⁺ circuit. We injected the pAAV-hSyn-DIO-hM3D(Gαq)-mCherry virus into the ACC of CR-Cre mice (Figure 2A). We activated this circuit using the DREADD agonist for 10 h, which was sufficient to increase proliferation of LV NSCs in the ventral SVZ near subep-ChAT⁺ neurons [12,13]. Whole-mount dissections from both hemispheres were stained for GABA, ChAT, and Mash1 (labels activated LV NSCs) (Figure 2B). We found more GABA⁺-Mash1⁺ NSCs on the ipsilateral (circuit activation) side compared to the contralateral (control) side (Figure 2C). Moreover, the intensity of GABA⁺ staining in cells near subep-ChAT⁺ neurons was significantly increased on the ipsilateral side (Figure 2D), demonstrating that the ACC-subep-ChAT⁺ circuit increases GABA in LV NSCs.

To further investigate how cholinergic signaling affects GABA in LV NSCs, in vitro experiments were conducted using cultured SVZ NSCs. Carbachol, a cholinergic agonist, was applied to half of the samples, with cells maintained in a quiescent state as described in Methods. After 24 h, both control and carbachol-treated samples were stained for GABA, epidermal growth factor receptor (EGFR), and DAPI to evaluate GABA modulation (Figure 2E). The results demonstrated that carbachol treatment increased GABA intensity in EGFR⁺ NSCs compared to controls (Figure 2F). LV NSCs within the ventral SVZ domain express GABA, especially in cells located near subep-ChAT⁺ neurons.

### 3.2. Expression of MAOB in LV NSCs Is Modulated by ACC-Subep-ChAT⁺ Circuit Activation

**Figure 3:**
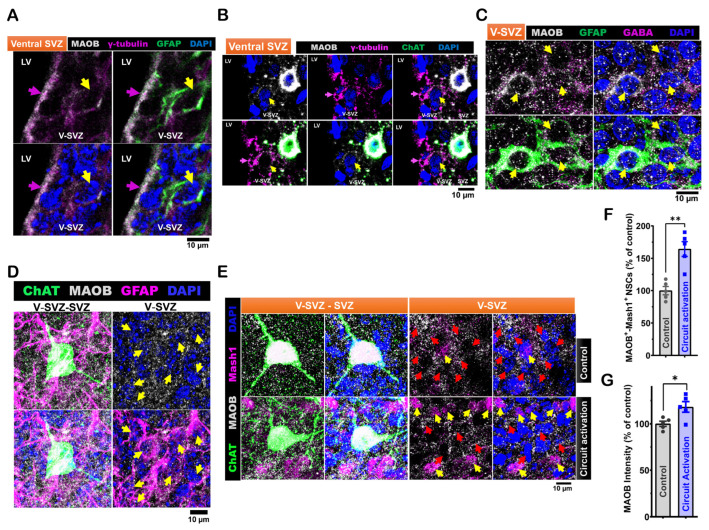
Expression of monoamine oxidase B (MAOB) in LV NSCs and its modulation by in vivo ACC-subep-ChAT^+^ circuit activity. (**A**). Representative immunofluorescence staining for MAOB (gray), GFAP (green), and γ-tubulin (purple) in coronal brain sections from P40 *C57BL/6J* mice (N = 3), specifically in the ventral V-SVZ. Yellow arrows indicate MAOB^+^-GFAP^+^-γ-tubulin^+^ NSCs in the ventral V-SVZ, while purple arrow indicates apical γ-tubulin⁺ NSC endings in the ventral SVZ. Scale bar = 10 μm. (**B**). Immunofluorescence staining for MAOB (gray), ChAT (green), and γ-tubulin (purple) in coronal brain sections of P40 *C57BL/6J* mice, showing the ventral V-SVZ adjacent to subep-ChAT^+^ neurons. Yellow arrows indicate MAOB^+^-γ-tubulin^+^ NSCs in the ventral V-SVZ, while purple arrow indicates apical γ-tubulin⁺ NSC endings in the ventral SVZ. Scale bar = 10 μm. (**C**). Immunofluorescence staining for MAOB (gray), GFAP (green), and GABA (purple) in SVZ wholemounts of P35 C57BL/6J mice. Yellow arrows indicate GABA^+^-GFAP^+^ cells in the ventral V-SVZ surrounding subep-ChAT^+^ neurons. Scale bar = 10 μm. (**D**). Immunofluorescence staining for MAOB (gray), ChAT (green), and GFAP (purple) in SVZ wholemounts of P35 C57BL/6J mice. Yellow arrows indicate MAOB^+^-GFAP^+^ cells in the ventral V-SVZ surrounding subep-ChAT^+^ neurons. Scale bar = 10 μm. (**E**). Immunofluorescence staining for MAOB (gray), ChAT (green), and Mash1 (purple) in contralateral and ipsilateral SVZ wholemounts of P35 *C57BL/6J* mice. The contralateral SVZ serves as a control, with no chemogenetic activation of the ACC-subep-ChAT^+^ circuit, and the ipsilateral SVZ is shown following chemogenetic activation, as described in Figure 2A. Yellow arrows indicate MAOB^+^-Mash1^+^ NSCs, while red arrows point to MAOB^+^ NSCs lacking Mash1 expression. Scale bar = 10 μm. (**F**). Quantification of MAOB^+^-Mash1^+^ NSCs relative to all MAOB^+^ NSCs per subep-ChAT^+^ neuron in contralateral and ipsilateral SVZ wholemounts from (**E**). ** *p* = 0.0030, t_4_ = 6.447, paired *t*-test. N = 5 *CR-Cre* mice. Each data point corresponds to the calculated mean percentage of MAOB⁺-Mash1⁺ NSCs as a subset of the total MAOB⁺ NSC population within a specified ROI. These ROIs were selected within the V-SVZ layer in proximity to subep-ChAT⁺ neurons (average from four subep-ChAT^+^ neurons per mouse). (**G**). Quantification of MAOB protein intensity relative to MAOB^+^ NSCs, measured per subep-ChAT^+^ neuron in contralateral and ipsilateral SVZ wholemounts from (**E**). * *p* = 0.0426, t_4_ = 2.935, paired *t*-test. N = 5 *CR-Cre* mice. Each data point in the analysis represents the mean percentage of MAOB protein intensity measured specifically within MAOB⁺ NSCs located in a defined ROI.

In coronal brain sections from P40 C57BL/6J mice, we found that GFAP^+^-γ-tubulin^+^ NSCs in the ventral ventricular-subventricular zone (V-SVZ) also expressed MAOB (Figure 3A). Moreover, γ-tubulin^+^ NSCs in this area, found next to subep-ChAT^+^ neurons, were likewise positive for MAOB (Figure 3B). In wholemount preparations of the SVZ from P35 C57BL/6J mice, we also found that GFAP^+^ NSCs in the ventral SVZ co-expressed GABA and MAOB (Figure 3C,D).

We then examined the modulation of MAOB protein expression in NSCs by the ACC-subep-ChAT^+^ circuit using a similar chemogenetic activation procedure as described above. SVZ wholemounts from both hemispheres (circuit activation and control) were stained for MAOB, ChAT, and Mash1. Circuit activation increased the number of MAOB^+^-Mash1^+^ NSCs (Figure 3E,F). Additionally, the intensity of MAOB protein in MAOB^+^ cells was markedly higher on the activated side compared to the control (Figure 3G). These findings show that MAOB is expressed in quiescent LV NSCs within the ventral SVZ and can be modulated by the activity of the ACC-subep-ChAT^+^ circuit.

### 3.3. LRRC8D Expression in LV NSCs and Its Functional Role in ACC-Subep-ChAT^+^ Circuit-Modulated Proliferation

**Figure 4:**
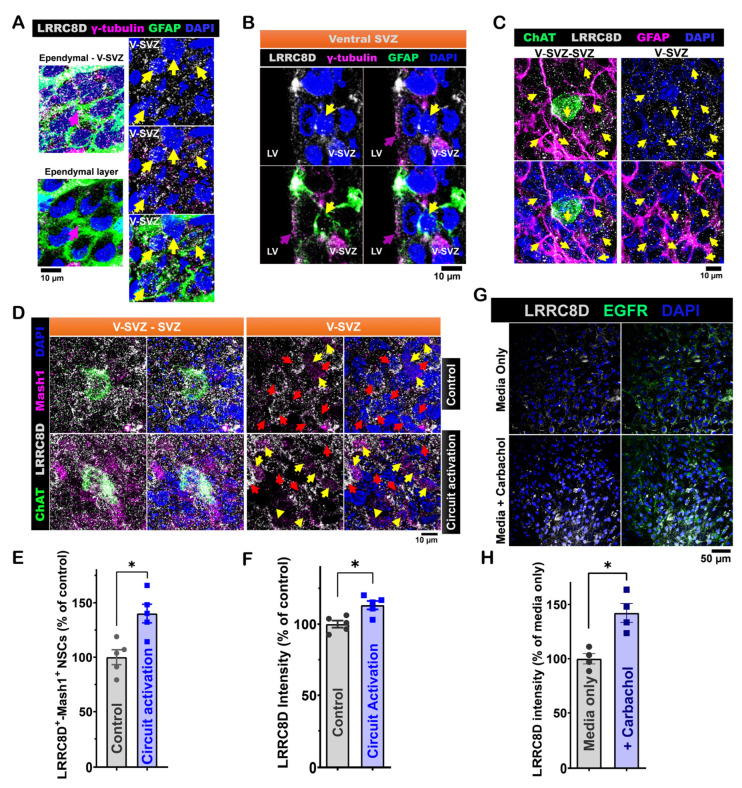
LRRC8D expression in ventral LV NSCs modulates their activity in response to ACC-subep-ChAT⁺ circuit signaling. (**A**). Immunofluorescence staining for LRRC8D (gray), GFAP (green), and γ-tubulin (purple) in the ventral SVZ from an SVZ wholemount of a P40 C57BL/6J mouse brain. Yellow arrows indicate LRRC8D⁺-GFAP⁺-γ-tubulin⁺ NSCs in the ventral V-SVZ, while purple arrows indicate apical endings of γ-tubulin⁺ NSCs in the ependymal layer. Scale bar = 10 μm. (**B**). Immunofluorescence staining for LRRC8D (gray), GFAP (green), and γ-tubulin (purple) in coronal brain sections of P40 C57BL/6J mice. Yellow arrows indicate LRRC8D⁺-GFAP⁺-γ-tubulin⁺ NSCs in the ventral V-SVZ, while purple arrows denote apical endings of γ-tubulin⁺ NSCs in the ventral SVZ. Scale bar = 10 μm. (**C**). Immunofluorescence staining for LRRC8D (gray), ChAT (green), and GFAP (purple) in SVZ wholemounts of P35 C57BL/6J mice. Yellow arrows indicate LRRC8D⁺-GFAP⁺ NSCs in the ventral V-SVZ surrounding subep-ChAT⁺ neurons. Scale bar = 10 μm. (**D**). Immunofluorescence staining for LRRC8D (gray), ChAT (green), and Mash1 (purple) in contralateral and ipsilateral SVZ wholemounts. Contralateral wholemounts serve as controls without chemogenetic activation of the ACC-subep-ChAT⁺ circuit, whereas ipsilateral wholemounts were analyzed following circuit activation, as described in Figure 2A. Yellow arrows indicate LRRC8D⁺-Mash1⁺ NSCs, while red arrows denote LRRC8D⁺ NSCs lacking Mash1 expression. Scale bar = 10 μm. (**E**). Quantification of LRRC8D⁺-Mash1⁺ NSCs relative to all LRRC8D⁺ NSCs per subep-ChAT⁺ neuron in contralateral and ipsilateral SVZ wholemounts from panel (**D**). * *p* = 0.0376, t₄ = 3.062, paired *t*-test. N = 5 CR-Cre mice. Each data point corresponds to the calculated mean percentage of LRRC8D⁺-Mash1⁺ NSCs as a subset of the total LRRC8D⁺ NSC population within a specified ROI. These ROIs were carefully delineated within the V-SVZ layer in proximity to subep-ChAT⁺ neurons (average from four subep-ChAT⁺ neurons per mouse). (**F**). Quantification of LRRC8D protein intensity relative to LRRC8D⁺ NSCs per subep-ChAT⁺ neuron in SVZ wholemounts from (**D**). * *p* = 0.0267, t_4_ = 3.422, paired *t*-test. N = 5 *CR-Cre* mice. Each data point in the analysis represents the mean percentage of LRRC8D protein intensity measured specifically within LRRC8D⁺ NSCs located in a defined ROI. ROIs were selected within the V-SVZ layer, focusing on areas immediately surrounding subep-ChAT⁺ neurons (averaged from four subep-ChAT⁺ neurons per mouse). (**G**). Immunofluorescence staining for LRRC8D (gray) and EGFR (green) in SVZ NSC cultures treated with control media or carbachol (15 μM) for 24 h in proliferation media. Scale bar, 50 μm. (**H**). Quantification of LRRC8D protein intensity/EGFR^+^ NSCs in control and carbachol-treated SVZ NSC cultures. * *p* = 0.0475, t_3_ = 3.249, paired *t*-test. N = 4 independent SVZ NSC cultures per group. Each data point represents the average percentage of LRRC8D protein intensity specifically within EGFR⁺ NSCs.

RNA-seq data from SVZ NSC cultures showed increased expression of the LRRC8D gene in LV NSCs [14]. We examined LRRC8D expression in LV NSCs by performing immunofluorescence staining on wholemount preparations from P40 C57BL/6J mouse brains. GFAP⁺-γ-tubulin⁺ NSCs were LRRC8D⁺ (Figure 4A). This finding was also confirmed in coronal brain sections of P40 C57BL/6J mice (Figure 4B). Using wholemounts from P35 C57BL/6J mice, we showed that GFAP⁺ NSCs, located in the ventral V-SVZ near subep-ChAT⁺ neurons, also expressed LRRC8D (Figure 4C).

To evaluate the functional role of LRRC8D in NSCs, we investigated its modulation by the activation of ACC-subep-ChAT⁺ circuit. Wholemounts of the ventral SVZ from both circuit-activated and control sides were stained for LRRC8D, ChAT, and Mash1 (Figure 4D). We found that circuit activation significantly increased the number of LRRC8D⁺-Mash1⁺ NSCs (Figure 4E). Additionally, circuit activation enhanced the protein intensity of LRRC8D compared to the control side (Figure 4F). When SVZ NSCs are treated with carbachol in vitro, there is an increase in LRRC8D compared to the control group (Figure 4G,H).

**Figure 5:**
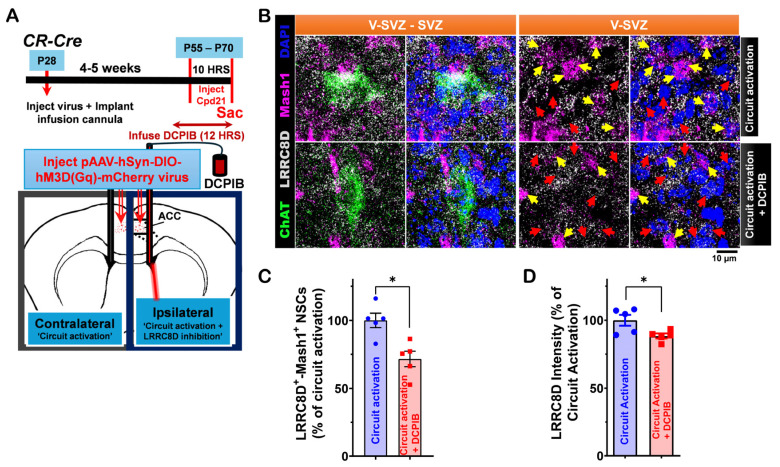
LRRC8D modulation of ventral LV NSCs proliferation activity induced by in vivo ACC-subep-ChAT^+^ circuitry. (**A**). Schematic representation (lower) and experimental design (upper) of in vivo chemogenetic activation for 10 h following injection of the pAAV-hSyn-DIO-hM3D(Gq)-mCherry virus into the ACC region and cannula implantation for DCPIB infusion into the LV region of P28 CR-Cre mice. (**B**). Immunofluorescence staining for LRRC8D (gray), ChAT (green), and Mash1 (purple) in contralateral SVZ wholemounts (control) and ipsilateral SVZ wholemounts after chemogenetic activation of the ACC-subep-ChAT⁺ circuit with DCPIB infusion, as illustrated in panel (**A**). Yellow arrows indicate LRRC8D⁺-Mash1⁺ NSCs, and red arrows denote LRRC8D⁺ NSCs lacking Mash1 expression. Scale bar = 10 μm. (**C**). Quantification of LRRC8D⁺-Mash1⁺ NSCs relative to all LRRC8D⁺ NSCs per subep-ChAT⁺ neuron in contralateral and ipsilateral SVZ wholemounts from panel (**A**). * *p* = 0.0467, t_4_ = 2.843, paired *t*-test. N = 5 CR-Cre mice. Each data point corresponds to the calculated mean percentage of LRRC8D⁺-Mash1⁺ NSCs as a subset of the total LRRC8D⁺ NSC population within a specified ROI. These ROIs were carefully delineated within the V-SVZ layer in proximity to subep-ChAT⁺ neurons (average from four subep-ChAT⁺ neurons per mouse). (**D**). Quantification of LRRC8D protein intensity relative to LRRC8D^+^ NSCs per subep-ChAT^+^ neuron in contralateral and ipsilateral SVZ wholemounts (panel **B**). * *p* = 0.0239, t_4_ = 3.544, paired *t*-test. N = 5 *CR-Cre* mice. Each data point in the analysis represents the mean percentage of LRRC8D protein intensity measured specifically within LRRC8D⁺ NSCs located in a defined ROI. These ROIs were selected within the V-SVZ layer, focusing on areas immediately surrounding subep-ChAT⁺ neurons (averaged from four subep-ChAT^+^ neurons per mouse).

Next, we assessed the impact of LRRC8D inhibition on this circuit-mediated modulation. We compared circuit-activated conditions with and without DCPIB, a selective LRRC8D inhibitor. CR-Cre mice were injected with pAAV-hSyn-DIO-hM3D(Gq)-mCherry virus into the ACC region, and a cannula was implanted to locally infuse DCPIB (5 µM) into the lateral ventricle (Figure 5A). Following the experiment, V-SVZ wholemounts were stained for LRRC8D, ChAT, and Mash1 (Figure 5B). We found a reduced number of LRRC8D⁺-Mash1⁺ NSCs on the circuit activation + DCPIB infusion side compared to the circuit activation only side (Figure 5C). We observed higher LRRC8D intensity on the circuit activation side compared to the circuit activation + DCPIB infusion side (Figure 5D). Selective LRRC8D inhibition may reduce the effects of circuit activation on LV NSC proliferation and LRRC8D protein expression.

### 3.4. SLC6A1 Expression and Its Role in Modulating ACC-Subep-ChAT^+^ Circuit Activity on LV NSCs in the Ventral SVZ

**Figure 6:**
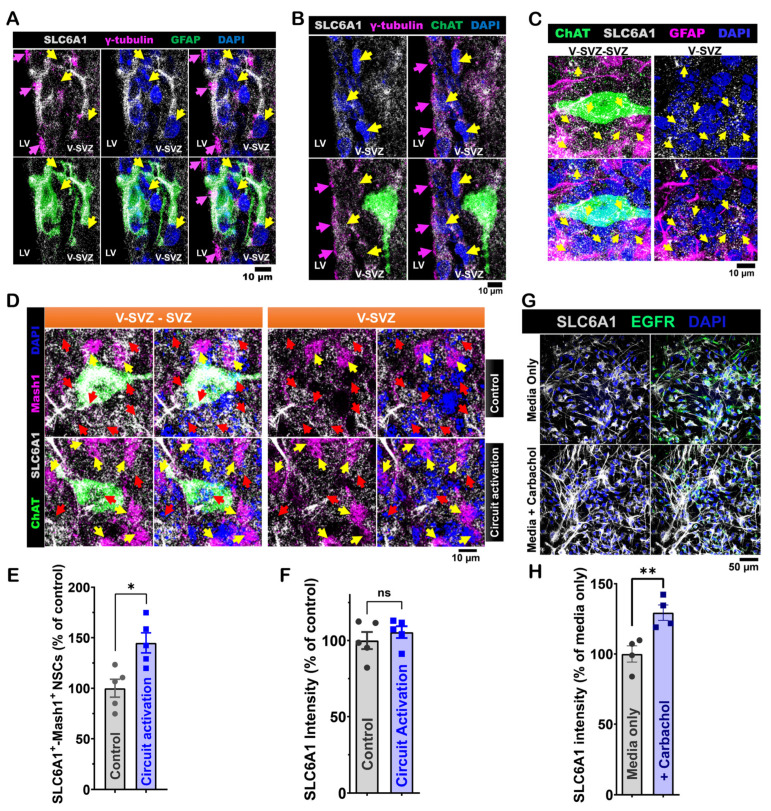
Ventral LV NSCs express SLC6A1, which is regulated by in vitro carbachol treatment. (**A**). Representative immunofluorescence staining for SLC6A1 (gray), GFAP (green), and γ-tubulin (purple) in the ventral V-SVZ of P40 *C57BL/6J* mouse brain coronal sections. Yellow arrows indicate SLC6A1^+^-GFAP^+^-γ-tubulin+ NSCs in the ventral V-SVZ. Purple arrows indicate γ-tubulin^+^ NSCs in the ventral SVZ. Scale bar = 10 μm. (**B**). Immunofluorescence staining for SLC6A1 (gray), ChAT (green), and γ-tubulin (purple) in the ventral V-SVZ adjacent to subep-ChAT^+^ neurons of P45 *C57BL/6J* mouse brain coronal sections. Yellow arrows indicate SLC6A1^+^-γ-tubulin^+^ NSCs in the ventral V-SVZ. Purple arrows indicate γ-tubulin^+^ NSCs in the ventral SVZ. Scale bar = 10 μm. (**C**). Immunofluorescence staining for SLC6A1 (gray), ChAT (green), and GFAP (purple) in SVZ wholemounts from *C57BL/6J* (P35) mice. Yellow arrows indicate SLC6A1^+^-GFAP^+^ cells in the ventral V-SVZ surrounding subep-ChAT^+^ neurons. Scale bar = 10 μm. (**D**). Immunofluorescence staining for SLC6A1 (gray), ChAT (green), and Mash1 (purple) in contralateral SVZ whole mounts without chemogenetic activation of the ACC-subep-ChAT^+^ circuit (control) and ipsilateral SVZ whole mounts after circuit activation (per Figure 2A). Yellow arrows indicate SLC6A1^+^-Mash1^+^ NSCs, while red arrows indicate NSCs expressing only SLC6A1. Scale bar = 10 μm. (**E**). Quantification of SLC6A1^+^-Mash1^+^ NSCs/SLC6A1^+^ NSCs per subep-ChAT^+^ neuron in contralateral and ipsilateral SVZ whole mounts from panel (**D**). * *p* = 0.0216, t_4_ = 3.657, paired *t*-test. N = 5 *CR-Cre* mice. Each data point corresponds to the calculated mean percentage of SLC6A1⁺-Mash1⁺ NSCs as a subset of the total SLC6A1⁺ NSC population within a specified ROI. These ROIs were carefully delineated within the V-SVZ layer in proximity to subep-ChAT⁺ neurons (averaged from four subep-ChAT^+^ neurons per mouse). (**F**). Quantification of SLC6A1 protein intensity/SLC6A1^+^ NSCs per subep-ChAT^+^ neuron in contralateral and ipsilateral SVZ wholemounts from panel (**D**). *p* = ns, t_4_ = 2.356, paired *t*-test. N = 5 CR-Cre mice. Each data point in the analysis represents the mean percentage of SLC6A1 protein intensity measured specifically within SLC6A1⁺ NSCs located in a defined ROI. These ROIs were selected within the V-SVZ layer, focusing on areas immediately surrounding subep-ChAT⁺ neurons (averaged from four subep-ChAT^+^ neurons per mouse). (**G**). Immunofluorescence staining for SLC6A1 (gray) and EGFR (green) in SVZ NSC cultures treated with control media or carbachol (15 μM) for 24 h in proliferation media. Scale bar, 50 μm. (**H**). Quantification of SLC6A1 protein intensity/EGFR+ NSCs in control and carbachol-treated SVZ NSC cultures. ** *p* = 0.0061, t₃ = 6.943, paired *t*-test. N = 4 independent SVZ NSC cultures per group. Each data point represents the average percentage of SLC6A1 protein intensity specifically within EGFR⁺ NSCs.

RNA sequencing data from SVZ NSC cultures shows moderate expression of the SLC6A1 gene [14]. SLC6A1 encodes GAT-1, which is essential for the reuptake of GABA. In coronal brain sections from P40 C57BL/6J mice, we found that quiescent GFAP^+^ and γ-tubulin^+^ NSCs in the ventral V-SVZ express SLC6A1 (Figure 6A). Similarly, in P45 mouse brain sections, γ-tubulin^+^ NSCs adjacent to subep-ChAT^+^ neurons also express SLC6A1 (Figure 6B). In SVZ wholemounts from P35 mice, GFAP^+^ NSCs surrounding subep-ChAT^+^ neurons in the ventral V-SVZ also express SLC6A1 (Figure 6C). In addition, ACC-subep-ChAT^+^ circuit activation increased the number of SLC6A1^+^ Mash1^+^ NSCs (Figure 6D,E). However, SLC6A1 intensity within SLC6A1^+^ cells was not affected (Figure 6F).

In SVZ NSC cultures, carbachol-treated NSCs showed higher SLC6A1 intensity, especially in the processes (Figure 6G,H). This finding suggests that cholinergic signaling increases GAT-1 in LV NSCs.

**Figure 7:**
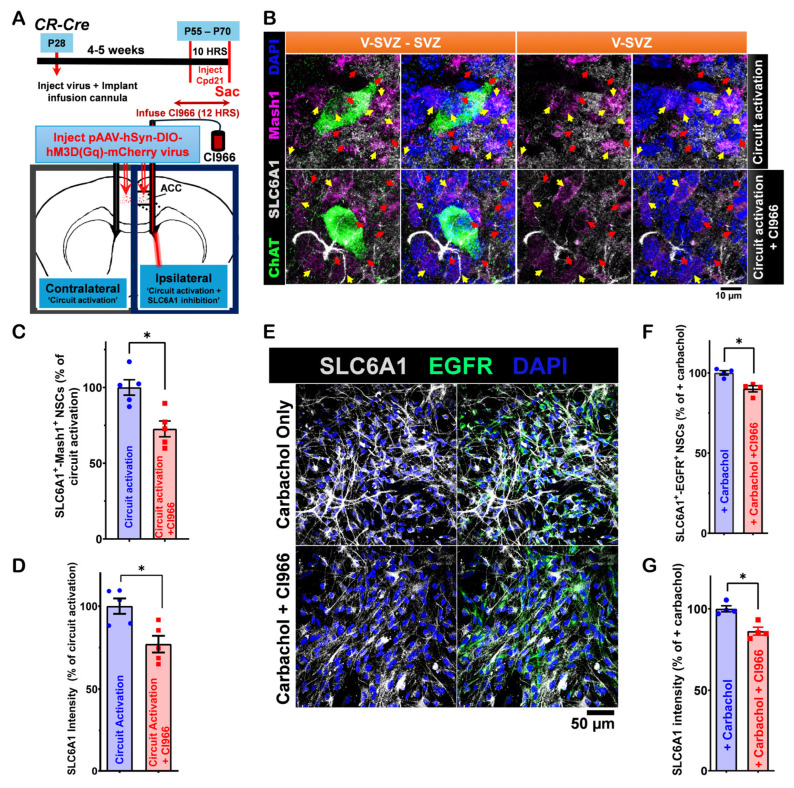
SLC6A1 modulation of ventral LV NSCs proliferation. (**A**). Schematic of the experimental design (upper) and of in vivo chemogenetic activation (lower). The procedure involved a 10 h activation following the injection of the pAAV-hSyn-DIO-hM3D(Gq)-mCherry virus into the ACC and cannula implantation for CI966 infusion into the lateral ventricle (LV) of P28 *CR-Cre* mice. (**B**). Immunofluorescence staining for SLC6A1 (gray), ChAT (green), and Mash1 (purple) in contralateral SVZ wholemounts after chemogenetic activation of the ACC-subep-ChAT^+^ circuit (control) and in ipsilateral SVZ wholemounts following circuit activation with CI966 infusion, as shown in panel (**A**). Yellow arrows indicate SLC6A1^+^-Mash1^+^ neural stem cells (NSCs), and red arrows indicate SLC6A1^+^ NSCs. Scale bar = 10 μm. (**C**). Quantification of SLC6A1^+^-Mash1^+^ NSCs relative to total SLC6A1^+^ NSCs per subep-ChAT^+^ neuron in contralateral and ipsilateral SVZ wholemounts (panel **B**). * *p* = 0.0395, t_4_ = 2.813, paired *t*-test. N = 5 *CR-Cre* mice. Each data point corresponds to the calculated mean percentage of SLC6A1⁺-Mash1⁺ NSCs as a subset of the total SLC6A1⁺ NSC population within a specified ROI. These ROIs were carefully delineated within the V-SVZ layer in proximity to subep-ChAT⁺ neurons (averaged across four subep-ChAT^+^ neurons per mouse). (**D**). Quantification of SLC6A1 protein intensity relative to SLC6A1^+^ NSCs per subep-ChAT^+^ neuron in contralateral and ipsilateral SVZ wholemounts (panel **B**). * *p* = 0.0482, t_4_ = 4.023, paired *t*-test. N = 5 *CR-Cre* mice. Each data point in the analysis represents the mean percentage of SLC6A1 protein intensity measured specifically within SLC6A1⁺ NSCs located in a defined ROI. These ROIs were selected within the V-SVZ layer, focusing on areas immediately surrounding subep-ChAT⁺ neurons (averaged from four subep-ChAT^+^ neurons per mouse). (**E**). Immunofluorescence staining for SLC6A1 (gray) and EGFR (green) in SVZ NSC cultures treated with carbachol (15 μM) or carbachol (15 μM) + CI966 (1.5 μM) after 24 h in the proliferation media. Scale bar = 50 μm. (**F**). Quantification of SLC6A1^+^-EGFR^+^ NSCs per well in SVZ NSC cultures treated with carbachol or carbachol + CI966. * *p* = 0.0452, t_3_ = 3.315, paired *t*-test. N = 4 independent SVZ NSC cultures per group. Each data point corresponds to the calculated mean percentage of SLC6A1^+^-EGFR^+^ NSCs. (**G**). Quantification of SLC6A1 protein intensity per EGFR+ NSCs in carbachol-treated and carbachol + CI966-treated SVZ NSC cultures. * *p* = 0.0208, t_3_ = 4.474, paired *t*-test. N = 4 independent SVZ NSC cultures per group. Each data point represents the average percentage of SLC6A1 protein intensity specifically within EGFR⁺ NSCs.

To assess how GAT-1 may modulate the effect of ACC-subep-ChAT^+^ circuit activation on LV NSC proliferation, we used chemogenetic activation as described above. On the ipsilateral side, GAT-1 activity was selectively inhibited using the GAT-1 inhibitor CI966 (1.5 µM) locally infused into the ventral SVZ (Figure 7A). Immunostaining for SLC6A1 (GAT-1), ChAT, and Mash1 revealed that SLC6A inhibition reduced the numbers of SLC6A1+-Mash1^+^ NSCs (Figure 7B,C). Additionally, SLC6A1 intensity was also reduced on the CI966-treated side (Figure 7D). These findings suggest that SLC6A1 (GAT-1) inhibition attenuates the influence of the ACC-subep-ChAT^+^ circuit on LV NSC proliferation and SLC6A1 expression.

Finally, cultures treated with both carbachol and CI966 exhibited a reduction in SLC6A1^+^ EGFR^+^ NSCs and lower SLC6A1 intensity compared to carbachol-only treated cultures (Figure 7E–G). Thus GAT-1 inhibition reduced the effect of cholinergic signaling on NSC proliferation. These results suggest that GAT-1 may also play a role in regulating ACC-subep-ChAT⁺ circuit-induced proliferation of LV NSCs within the ventral SVZ domain.

## 4. Discussion

Our previous work showed that the ACC-subep-ChAT^+^ circuit can activate quiescent NSCs and promote proliferation via cholinergic signaling [13]. In this study, we investigated whether LV NSCs can also send a negative feedback signal to regulate the ACC-subep-ChAT^+^ circuit and in turn affect proliferation of LV NSCs surrounding subep-ChAT^+^ neurons. Previous work showed that subep-ChAT^+^ neurons can be inhibited by GABAergic signaling [12]. Here we examined the role of GABA signaling in this network.

Our findings suggest that LV NSCs surrounding subep-ChAT^+^ neurons can produce and potentially release GABA (Figure 1). Since these GABA-producing LV NSCs is influenced by the ACC-subep-ChAT^+^ circuit, GABA may play a role in regulating the subep-ChAT^+^ neurons and NSC activity, in response to circuit activation and cholinergic signaling (Figure 2).

We investigated the potential of LV NSCs to synthesize GABA by examining whether MAOB, which is highly expressed in both quiescent and active states of LV NSCs, plays a role in GABA production. We found that the ACC-subep-ChAT^+^ circuit modulates the activity of the MAOB protein in LV NSCs (Figure 3). Previous studies have identified the putrescine degradation pathway as a biosynthetic mechanism for GABA in astrocytes, with MAOB acting as a key enzyme in this process [16,28]. Given the glia-like properties of LV NSCs, it is possible that they also use similar mechanisms to produce GABA.

RNA-seq analysis shows that LV NSCs do not express vesicular genes associated with GABA release. Instead, we identified high expression of several LRRC8 subunits, including LRRC8D, LRRC8A, LRRC8C, and LRRC8B, with LRRC8D levels notably reduced following carbachol treatment [14]. LRRC8 subunits form heteromeric VRACs that transport neurotransmitters like glutamate and GABA [21,29]. Our results suggest that LRRC8D may regulate the activity of LV NSCs (Figure 4). Moreover, selective inhibition of LRRC8D within the ventral SVZ resulted in a reduction in LV NSC proliferation (Figure 5).

Selective inhibition of LRRC8D (VRAC) reduces LV NSC proliferation via the ACC-subep-ChAT^+^ circuit (Figure 5B,C), suggesting that intracellular GABA accumulation may result from impaired GABA release into the extracellular space. LRRC8D inhibition also reduces its expression in LV NSCs (Figure 5D). GABA accumulation could be a response to sustained activation of the ACC-subep-ChAT^+^ circuit. One possibility is that circuit activation promotes GABA synthesis and reuptake from the extracellular space into LV NSCs, followed by its release via LRRC8D. Thus, inhibiting LRRC8D disrupts this process and increases intracellular GABA levels, which may impair NSC proliferation associated with circuit activation. Moreover, LRRC8D plays a crucial role in chloride homeostasis and cell volume regulation. VRAC activation, typically triggered by hypotonic stress, facilitates chloride efflux to restore cellular osmotic balance. Given its role in anion transport, another possibility is that inhibiting LRRC8D can also reduce chloride efflux, thus preventing LV NSC depolarization and proliferation. Further research is needed to elucidate the precise molecular mechanisms affected by intracellular GABA accumulation in LV NSCs or changes in chloride transport, and to determine how these alterations influence circuit activity governing NSC proliferation.

We also investigated the capacity of LV NSCs to reuptake GABA from the extracellular space. RNA-seq analysis of SVZ NSCs revealed that both quiescent and active NSCs express the Slc6a1 gene [14], which encodes GAT-1, a key transporter for GABA. Dysfunction or mutations in SLC6A1 have been linked to neurodevelopmental disorders, in which disrupted GABAergic signaling is associated with developmental delays, epilepsy, autism spectrum disorders, and motor dysfunction [30].

We found that LV NSCs next to subep-ChAT^+^ neurons express SLC6A1 (GAT-1), which transports GABA from the extracellular to intracellular space. Inhibiting GAT-1 is therefore expected to increase GABA signaling in the extracellular space. Indeed, we found that pharmacological inhibition of GAT-1 with CI966 significantly reduced the impact of the ACC-subep-ChAT^+^ circuit on LV NSC proliferation. This finding demonstrates the functional significance of GABA transport in negative feedback signaling.

Our results reveal differences in SLC6A1 protein intensity between in vivo (Figure 6D–F; control vs. circuit activation) and in vitro (Figure 6G,H; control vs. carbachol treatment) experiments. While in vivo experiments showed no significant change in SLC6A1 protein intensity following circuit activation, in vitro experiments demonstrated a significant increase after carbachol treatment. This discrepancy may be attributed to differences in imaging conditions.

The in vitro experiment provides a clear visualization of NSC somas and their processes, showing that SLC6A1 is highly concentrated in LV NSC processes and can be accurately quantified. In contrast, the imaging in the in vivo experiment is limited by the depth of the V-SVZ layer, where LV NSCs reside, as well as the complex distribution of their processes. This makes it difficult to capture a comprehensive image of the entire V-SVZ layer without losing critical details, such as SLC6A1 protein intensity in NSC processes, ultimately affecting the accuracy of SLC6A1 intensity measurements in in vivo experiments.

Additionally, our RNA-seq data revealed upregulation of the SLC6A1 gene following carbachol treatment [14], in accord with the observed increase in SLC6A1 protein levels upon cholinergic activation in LV NSCs. This suggests that LV NSCs may upregulate SLC6A1 protein to facilitate GABA uptake, potentially contributing to a negative feedback mechanism that inhibits cholinergic signaling in response to ACC-subep-ChAT^+^ circuit activation.

Selective inhibition of SLC6A1 receptors significantly attenuates the ACC-subep-ChAT^+^ circuit-induced increase in SLC6A1 protein expression (Figure 7), suggesting an indirect regulatory mechanism. Blocking SLC6A1 disrupts GABA reuptake into LV NSCs, potentially reducing intracellular GABA concentrations. This reduction may impair the activation of key signaling pathways required for SLC6A1 upregulation, thereby influencing downstream neurophysiological processes. However, the precise molecular interactions underlying this effect remain unclear. Further investigation is necessary to elucidate the mechanistic link between SLC6A1 inhibition, intracellular GABA dynamics, and the regulatory pathways governing SLC6A1 expression, as well as their impact on LV NSC proliferation and activity.

Direct assessment of GABA release from LV NSCs onto subep-ChAT⁺ neurons following their activation remains challenging due to the lack of specific experimental tools. Previous findings have shown that local GABAergic interneurons release GABA, inhibiting subep-ChAT⁺ neurons and thereby influencing the ACC-subep-ChAT⁺ circuit and LV NSC proliferation [12]. However, it is at present not possible to measure GABA signaling in subep-ChAT⁺ neurons in response to GABA release from LV NSCs. Blocking GABA receptors on subep-ChAT⁺ neurons do not exclusively affect GABA signaling from LV NSCs, since subep-ChAT⁺ neurons are also modulated by GABAergic interneurons.

## 5. Conclusions

**Figure 8:**
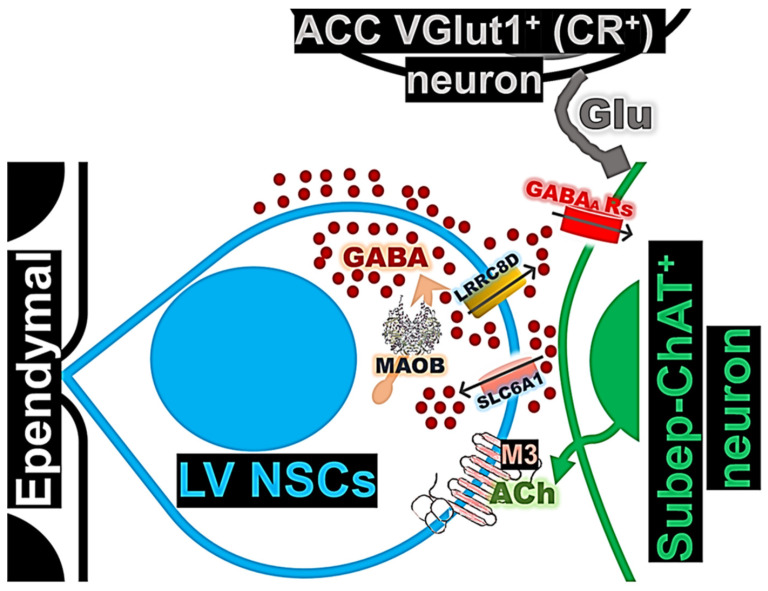
A model depicts GABA signaling in the LV NSCs, highlighting the regulation of ACC-subep-ChAT^+^ circuit activity and its influence on LV NSC proliferation.

Our findings underscore the critical role of GABA signaling in LV NSCs, specifically in regulating subep-ChAT^+^ activity and orchestrating the dynamic interactions between the ACC-subep-ChAT^+^ circuit and LV NSCs. The proposed model suggests that GABAergic signaling from NSCs acts as a negative feedback mechanism, influencing both circuit activation and ultimately stem cell dynamics within the ventral SVZ. It is possible that GABA is produced within LV NSCs through the MAOB enzymatic pathway and subsequently transported to the extracellular space via LRRC8D (Figure 8). Additionally, our model reveals that LV NSCs express SLC6A1 (GAT-1) in response to ACC-subep-ChAT^+^ circuit activation, which facilitates the reuptake of extracellular GABA and enables LV NSCs to regulate their proliferation and activity. Further investigation is required to elucidate the precise molecular mechanisms and broader implications of these processes in neurodevelopmental and neurodegenerative disorders. While the regulatory mechanisms outlined here are triggered by chemogenetic circuit stimulation, additional research is necessary to determine whether similar mechanisms occur during endogenous activation of the niche. Furthermore, exploring potential negative feedback mechanisms for circuit activation of NSCs in other regions, may provide valuable insights into other types of neurogenesis, such as neurogenesis in the dentate gyrus of the postnatal and adult hippocampus.

## Figures and Tables

**Table 1:** Functional characterization of markers used for quiescent and activated states of NSCs and Subep-ChAT^+^ neurons in this study.

Marker	Marker Function	Cellular Localization	Cell Type
γ-Tubulin	Essential for mitotic spindle organization	Cytoplasmic and centrosomal, predominantly located at the apical region of quiescent LV NSCs	Quiescent LV NSCs
GFAP	Astrocyte and neural stem cell (NSC) marker, indicative of glial properties	Mainly localized in the cytoplasm, associated with cytoskeletal structures	Quiescent LV NSCs, Activated LV NSCs
EGFR	Epidermal growth factor receptor, promotes NSC self-renewal and proliferation	Membrane-bound	Quiescent LV NSCs, Activated LV NSCs
Mash1 (Ascl1)	Transcription factor that promotes the early stages of neurogenesis	Nuclear	Activated LV NSCs
GABA	Inhibitory neurotransmitter	Cytoplasmic, extracellular	Quiescent LV NSCs, Activated LV NSCs
MAOB	Enzyme involved in GABA synthesis	Mitochondrial membrane	Quiescent LV NSCs, Activated LV NSCs
LRRC8D	Volume-regulated anion channel component, mediates transport	Membrane-bound	Quiescent LV NSCs, Activated LV NSCs
SLC6A1 (GAT-1)	GABA transporter, regulates extracellular GABA levels	Membrane-bound proteins and processes of LV NSCs	Quiescent LV NSCs, Activated LV NSCs
ChAT	Catalyzes acetylcholine (ACh) synthesis	Cytoplasmic	Subep-ChAT^+^ Neurons
ACh	Neurotransmitter involved in LV NSC activation	Extracellular	Subep-ChAT^+^ Neurons

## Data Availability

The original contributions presented in this study are included in the article/Supplementary Material. Further inquiries can be directed to the corresponding authors.

## Supplementary Materials

The following supporting information can be downloaded at: https://www.mdpi.com/article/10.3390/cells14060426/s1, Figure S1 GABA intensity in LV NSCs surrounding subep-ChAT^+^ neurons between P30 and P55 C57BL/6 mice; Figure S2: GABA intensity in LV NSCs surrounding subep-ChAT⁺ neurons across hemispheres at P55

## Abbreviations

The following abbreviations are used in this manuscript:

LV NSCs	lateral ventricular neural stem cells
GABA	Gamma-aminobutyric acid
ACC-subep-ChAT+	Anterior cingulate cortex-subependymal-ChAT+
MAOB	Monoamine oxidase B
GAT-1	GABA Transporter-1
VRACs	Volume-regulated anion channels
