# Supplementary material for: Lateral Ventricular Neural Stem Cells Provide Negative Feedback to Circuit Activation Through GABAergic Signaling
Source: Cells. 2025 Mar 13;14(6):426. doi: 10.3390/cells14060426 (PMC11940892; doi:10.3390/cells14060426)
Supplement: Supplementary file 1 [file cells-14-00426-s001.zip › cells-3439510-supplementary.pdf]

Supplementary materials

Lateral Ventricular Neural Stem Cells Provide Negative Feedback to Circuit Activation Through GABAergic Signaling

Moawiah M. Naffaa [1,2,*] and Henry H. Yin [1,3,*]

Figure S1: GABA intensity in LV NSCs surrounding subep-ChAT+ neurons between P30 and P55 C57BL/6 mice.

Figure S2: GABA intensity in LV NSCs surrounding subep-ChAT$^+$ neurons across hemispheres at P55.

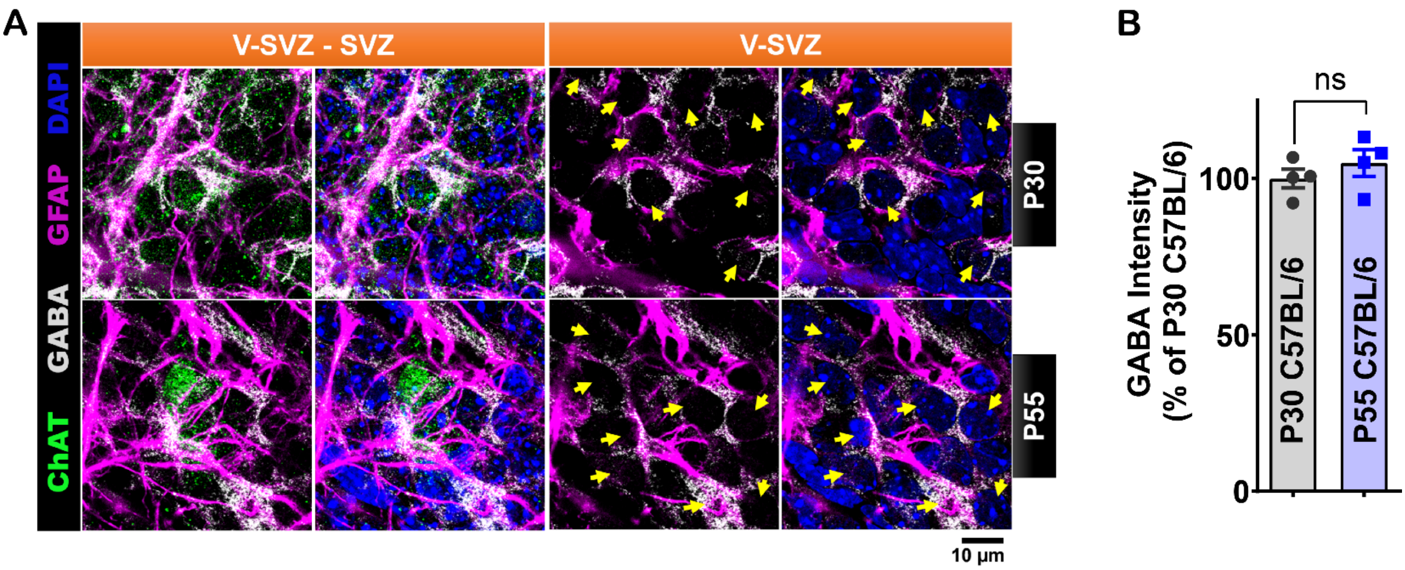

Figure S1: GABA intensity in LV NSCs surrounding subep-ChAT⁺ neurons between P30 and P55 C57BL/6 mice. (**A**). Immunofluorescence staining for GABA (gray), ChAT (green), and GFAP (purple) in SVZ whole-mount preparations of *C57BL/6* mice at P30 (top) and P55 (bottom). Yellow arrows indicate GABA⁺-GFAP⁺ cells in the ventral V-SVZ surrounding subep-ChAT⁺ neurons. Scale bar = 10 µm. Images are representative of four mice. (**B**). Quantification of GABA intensity relative to GABA⁺ NSCs per subep-ChAT⁺ neuron in SVZ wholemounts from (**A**). *P = 0.3379, t3 = 1.138, paired t-test. N = 4 *C57BL/6* mice. Each data point in the analysis represents the mean percentage of GABA intensity measured specifically within GABA⁺ NSCs located in a defined ROI. ROIs were selected within the V-SVZ layer, in areas immediately surrounding subep-ChAT⁺ neurons (averaged from four subep-ChAT⁺ neurons per mouse).

Supplementary materials

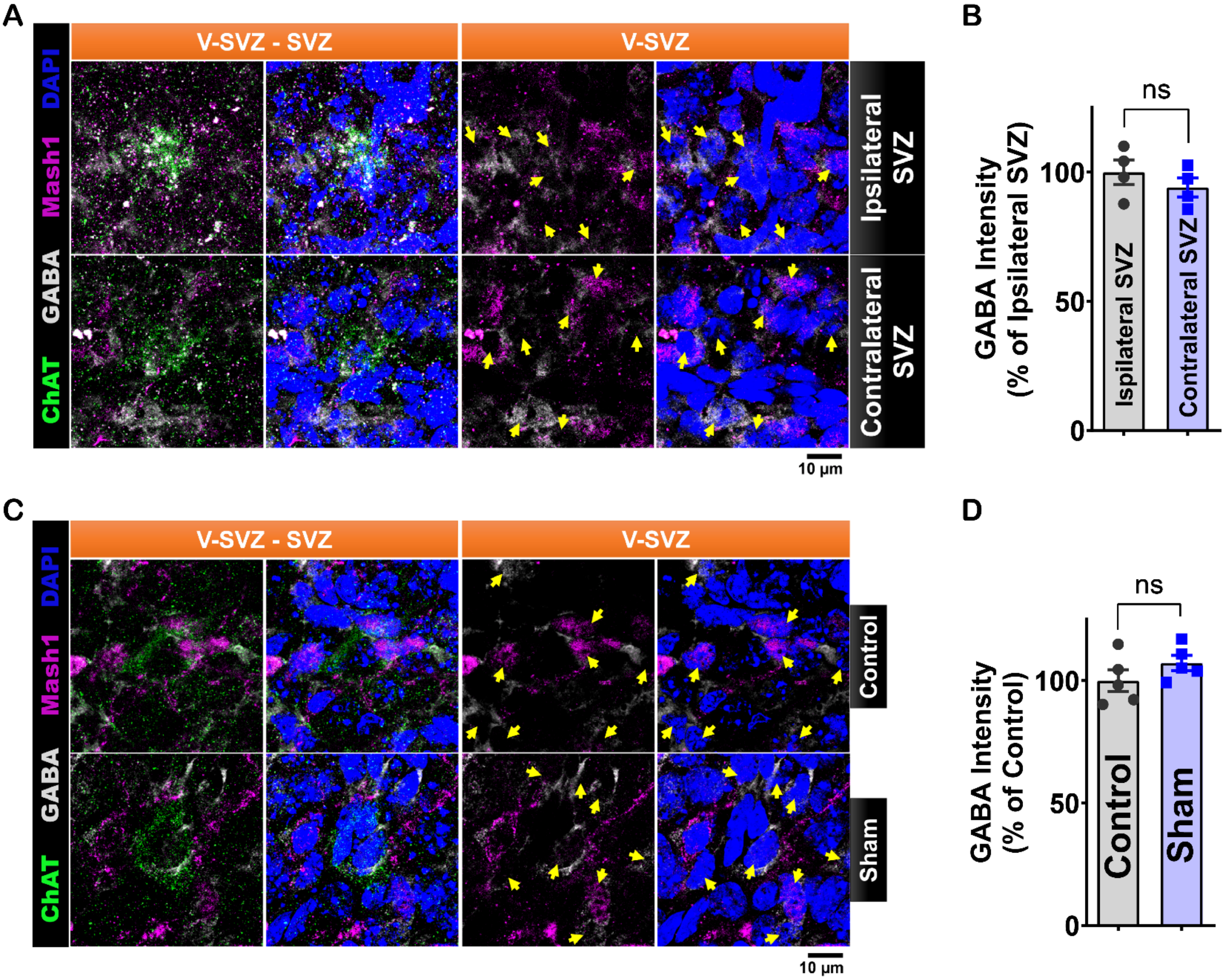

Figure S2: GABA intensity in LV NSCs surrounding subep-ChAT⁺ neurons across hemispheres at P55. (**A**). Immunofluorescence staining for GABA (gray), ChAT (green), and Mash1 (purple) in ipsilateral and contralateral SVZ whole-mount preparations of P55 *C57BL/6* mice. Yellow arrows indicate GABA⁺ cells in the ventral V-SVZ surrounding subep-ChAT⁺ neurons. Scale bar = 10 µm. Images are representative of four mice. (**B**). Quantification of GABA intensity relative to GABA⁺ per subep-ChAT⁺ neuron in SVZ wholemounts from (A). *P = 0.2295, t3 = 1.505, paired t-test. N = 4 *C57BL/6* mice. Each data point in the analysis represents the mean percentage of GABA intensity measured specifically within GABA⁺ cells located in a defined ROI. ROIs were selected within the V-SVZ layer, in areas immediately surrounding subep-ChAT⁺ neurons (averaged from four subep-ChAT⁺ neurons per mouse). (**C**). Immunofluorescence staining for GABA (gray), ChAT (green), and Mash1 (purple) in SVZ

whole-mount preparations of P55 *CR-Cre* mice (Control; upper) and (Sham; lower). The Control side received no injection; The Sham hemisphere was injected with pAAV-hSyn-DIO-hM3D(Gαq)-mCherry virus at P28. Yellow arrows indicate $GABA^+$ cells in the ventral V-SVZ surrounding subep-$ChAT^+$ neurons. Scale bar = 10 μm. (**D**). Quantification of GABA intensity relative to $GABA^+$ per subep-$ChAT^+$ neuron in SVZ wholemounts from (**A**). *P = 0.2754, t4 = 1.263, paired t-test. N = 5 *CR-Cre* mice for the Control group and N = 5 *CR-Cre* mice for the Sham group. Each data point in the analysis represents the mean percentage of GABA intensity measured specifically within $GABA^+$ cells located in a defined ROI. ROIs were selected within the V-SVZ layer, in areas immediately surrounding subep-$ChAT^+$ neurons (averaged from five subep-$ChAT^+$ neurons per mouse).
